# An immune-inflammatory dysregulation score for risk stratification of 28-day all-cause mortality in critically ill patients with severe community-acquired pneumonia

**DOI:** 10.3389/fmed.2026.1923412

**Published:** 2026-09-01

**Authors:** Shulan Zhou, Bin Fu, Ziyi Huang, Hanqi Wang, Yongjian Pei, Chen Chen, Tong Zhou, Zengli Zhang

**Affiliations:** Department of Respiratory and Critical Care Medicine, The Second Affiliated Hospital of Soochow University, Suzhou, China

**Keywords:** immune-inflammatory dysregulation score, nomogram, prognosis, risk prediction, severe community-acquired pneumonia

## Abstract

**Background:**

Severe community-acquired pneumonia (SCAP) is associated with high mortality and substantial heterogeneity in host immune responses among critically ill patients. Conventional prognostic scores primarily reflect comorbidity burden and organ dysfunction but do not directly capture immune dysregulation.

**Methods:**

This retrospective cohort study included 223 critically ill patients with SCAP admitted to the respiratory intensive care unit (RICU). Six prespecified immune-inflammatory biomarkers were evaluated as candidate predictors. The Immune-Inflammatory Dysregulation Score (IIDS) was developed using bootstrap-based stability selection (1,000 bootstrap resamples) followed by multivariable logistic regression. The discriminative performance of IIDS was compared with conventional clinical severity scores, and its incremental prognostic value was assessed using sequential prediction models. A final prediction model incorporating IIDS was internally evaluated using bootstrap optimism correction and presented as a nomogram.

**Results:**

IIDS comprised CD4^+^ T-cell count, monocyte count, and the neutrophil-to-lymphocyte ratio. IIDS showed moderate apparent discrimination for 28-day mortality (AUC: 0.721; 95% CI: 0.654–0.787). Adding IIDS to the CCI + SOFA model increased the apparent AUC from 0.773 to 0.819 (DeLong *P* = 0.014) and the bootstrap optimism-corrected AUC from 0.767 to 0.809. Optimism-corrected reclassification estimates were imprecise, with confidence intervals for NRI and IDI including zero. The final model showed close agreement between predicted and observed risks after optimism correction and provided potential net clinical benefit across the evaluated threshold-probability range.

**Conclusions:**

IIDS provided prognostic information complementary to conventional clinical predictors and improved apparent discrimination when incorporated into a sequential prediction model. Independent external validation is required before clinical implementation.

## 1 Introduction

Severe community-acquired pneumonia (SCAP) frequently necessitates intensive care and continues to carry substantial short-term mortality despite advances in antimicrobial therapy and organ support. Mortality varies across settings and patient populations but remains particularly high among patients requiring mechanical ventilation or other forms of intensive organ support (1–3). Timely identification of patients at increased risk of death is therefore central to treatment escalation, risk-adapted monitoring, and the appropriate allocation of critical care resources.

Poor outcomes in SCAP reflect a dysregulated host response rather than an unchecked inflammatory response alone (4–7). Hyperinflammation may coexist with impaired antimicrobial immunity, involving lymphocyte depletion, altered T-cell responses, and dysfunctional myeloid-cell states (4–8). Clinical studies have linked these immune alterations, particularly lymphopenia and depletion of CD4^+^ T-cell subsets, to greater disease severity, delayed recovery, and mortality in community-acquired pneumonia (4–7). More recently, single-cell transcriptomic profiling of bacterial pneumonia has revealed extensive remodeling of the peripheral immune landscape, including T-cell exhaustion and the expansion of immunosuppressive myeloid-cell populations (8). Together, these observations provide a biological rationale for incorporating circulating immune-cell measures into prognostic assessment in SCAP.

Current prognostic tools capture different aspects of clinical severity. The Charlson Comorbidity Index (CCI) quantifies comorbidity burden (9), whereas the Sequential Organ Failure Assessment (SOFA) score measures acute organ dysfunction (10, 11). The Pneumonia Severity Index (PSI) (12) and CURB-65 score (13) combine demographic, clinical, and laboratory variables to estimate pneumonia severity and mortality risk. Although clinically useful, these instruments do not directly quantify host immune dysregulation and may therefore overlook biologically distinct immune phenotypes within SCAP.

Circulating immune and inflammatory measures may provide information that is not explicitly represented in conventional clinical scores. Reduced CD4^+^ T-cell counts and lymphopenia have been associated with greater severity and mortality in community-acquired pneumonia (4–7), while monocyte alterations may reflect disruption of innate immune responses (8). The neutrophil-to-lymphocyte ratio (NLR) integrates changes in circulating neutrophils and lymphocytes and has been associated with adverse outcomes in CAP, although its incremental prognostic value beyond established severity scores has varied across studies (14–16). No single marker, however, adequately represents the interacting adaptive, innate, and inflammatory components of immune dysregulation.

On this basis, we developed the Immune-Inflammatory Dysregulation Score (IIDS), a composite signature derived from routinely available measures of adaptive immunity, innate immunity, and systemic inflammation. IIDS was intended to complement, rather than replace, established clinical predictors by capturing an additional dimension of host immune status.

We therefore aimed to develop and internally evaluate an immune-informed model for predicting 28-day mortality among patients with SCAP admitted to the RICU. We derived IIDS using bootstrap-based stability selection and assessed its incremental prognostic contribution beyond conventional clinical scores using sequential models. The final model was internally evaluated using bootstrap resampling and presented as a nomogram for individualized estimation of 28-day mortality.

## 2 Materials and methods

### 2.1 Study design and population

This retrospective cohort study was conducted in the Respiratory Intensive Care Unit (RICU) of The Second Affiliated Hospital of Soochow University. Consecutive adults admitted to the RICU with severe community-acquired pneumonia (SCAP) between January 2022 and December 2024 were screened for eligibility.

The diagnosis of community-acquired pneumonia and classification of SCAP were based on the 2019 American Thoracic Society/Infectious Diseases Society of America (ATS/IDSA) clinical practice guideline (1). SCAP was defined by the presence of at least one major criterion or three or more minor criteria. The major criteria were septic shock requiring vasopressor therapy and respiratory failure requiring invasive mechanical ventilation. The minor criteria were a respiratory rate ≥30 breaths/min, a PaO_2_/FiO_2_ ratio ≤250, multilobar infiltrates, confusion or disorientation, a blood urea nitrogen level ≥20 mg/dL, leukopenia (white blood cell count <4,000 cells/µL), thrombocytopenia (platelet count <100,000 cells/µL), hypothermia (core temperature <36 °C), and hypotension requiring aggressive fluid resuscitation. The diagnosis was independently confirmed by two senior respiratory physicians on the basis of clinical presentation, laboratory findings, microbiological results, and chest imaging. Patients with hospital-acquired pneumonia, ventilator-associated pneumonia, healthcare-associated pneumonia, or aspiration pneumonia were not eligible.

Patients were eligible if they were aged ≥18 years, were admitted to the RICU with SCAP, and had peripheral blood lymphocyte-subset measurements and routine laboratory results obtained within 24 h of RICU admission. All variables required to calculate IIDS and conventional severity scores and to determine the study outcome also had to be available.

Patients were excluded if they had human immunodeficiency virus infection or if their 28-day outcome status was unavailable. Patients with malignancy, autoimmune disease, previous chemotherapy or radiotherapy, long-term corticosteroid exposure, immunosuppressive therapy, or other immunocompromising conditions were retained to reflect the patient population encountered in routine RICU practice. The potential influence of baseline immunosuppression and immunomodulatory therapy on model performance was examined in sensitivity analyses.

The primary outcome was death from any cause within 28 days after RICU admission.

The study was approved by the Institutional Review Board of The Second Affiliated Hospital of Soochow University (approval no. JD-HG-2025-100). The requirement for written informed consent was waived because the study retrospectively analyzed anonymized clinical data.

This study was reported in accordance with the Transparent Reporting of a multivariable prediction model for Individual Prognosis or Diagnosis (TRIPOD) statement (17).

### 2.2 Data collection and candidate variables

Baseline demographic characteristics, comorbidities, laboratory findings, microbiological results, treatment variables, and 28-day outcome data were retrieved from the electronic medical records. Conventional severity scores, including the Charlson Comorbidity Index (CCI), Sequential Organ Failure Assessment (SOFA) score, Pneumonia Severity Index (PSI), and CURB-65 score, were calculated using clinical and laboratory data obtained within 24 h of RICU admission.

Laboratory measurements included complete blood counts, inflammatory biomarkers, serum biochemical indices, coagulation parameters, arterial blood gas measurements, and lactate concentrations. Absolute counts of lymphocyte subsets, including total T cells, CD4^+^ T cells, CD8^+^ T cells, natural killer (NK) cells, and B cells, were quantified by flow cytometry in accordance with the manufacturer's instructions and standard institutional laboratory procedures. Monocyte counts and the neutrophil-to-lymphocyte ratio (NLR) were obtained from routine hematological testing performed on admission.

Clinical management variables included mechanical ventilation, vasopressor therapy, and corticosteroid treatment after RICU admission. Microbiological findings included the detection of bacterial, viral, fungal, and atypical pathogens.

Six immune-inflammatory biomarkers were prespecified as candidate variables for development of the Immune-Inflammatory Dysregulation Score (IIDS): CD4^+^ T-cell count, CD8^+^ T-cell count, NK-cell count, B-cell count, monocyte count, and NLR. These biomarkers were selected to represent complementary components of adaptive immunity, innate immune responses, and systemic inflammation.

Before statistical analysis, all extracted data were independently verified by two investigators. The internal consistency of laboratory variables was assessed by cross-checking flow-cytometry measurements and routine laboratory results against the original medical records. Among three potentially inconsistent flow-cytometry records identified during data verification, two data-entry or transcription errors were corrected according to the source laboratory reports. For the remaining record, the inconsistency was present in the source report and could not be reliably corrected; the source-recorded values were retained in the primary analysis, and exclusion of this record was assessed in a sensitivity analysis. Implausible or extreme laboratory values were also re-verified against the source records.

### 2.3 Construction of the immune-inflammatory dysregulation score

Six prespecified immune-inflammatory biomarkers, comprising CD4^+^ T-cell count, CD8^+^ T-cell count, NK-cell count, B-cell count, monocyte count, and the neutrophil-to-lymphocyte ratio (NLR), were considered as candidate predictors for IIDS construction. Because several biomarkers had markedly right-skewed distributions and a small number of monocyte and B-cell counts were recorded as zero, all candidate biomarkers were transformed using the natural logarithm after adding one, defined as log1*p*(*x*) = ln(*x* + 1). This transformation retained observations with zero values while reducing distributional skewness.

Pairwise correlations among the candidate biomarkers were assessed using Spearman correlation coefficients. Restricted cubic spline analyses were then performed to examine the functional relationship between each biomarker and 28-day all-cause mortality. Variable selection was performed using bootstrap stability selection with 1,000 bootstrap resamples. Candidate biomarkers with a selection frequency ≥0.80 were retained and entered into a multivariable logistic regression model to derive IIDS.

The final IIDS was calculated as the linear predictor from the multivariable logistic regression model:

$$\mathrm{IIDS}=-0.5475\times \log\;1p(\mathrm{CD}4^{+\;}T-\mathrm{cell}\;\mathrm{count})-1.8078\times \log\;1p(\mathrm{monocyte}\;\mathrm{count})+0.5915\times \log\;1p(\mathrm{NLR})$$

In the study cohort, IIDS ranged from −4.82 to 0.83, with a median of −1.88 (interquartile range, −2.33 to −1.16). IIDS was analyzed as a continuous predictor without categorization or standardization.

For subsequent clinical prediction modeling, IIDS was incorporated as a continuous predictor into sequential multivariable logistic regression models together with established clinical severity indices; the individual immune biomarkers were not re-entered into these models. This prespecified two-stage strategy was used to construct a parsimonious immune signature while avoiding redundancy among correlated immune variables.

### 2.4 Development and internal evaluation of sequential prediction models

Receiver operating characteristic (ROC) curves were generated to evaluate the apparent discriminative performance of IIDS and the conventional clinical severity scores. To assess the incremental prognostic contribution of IIDS, three prespecified sequential logistic regression models were developed: Model 1 included CCI; Model 2 included CCI and SOFA; and Model 3 included CCI, SOFA, and IIDS.

Model performance was evaluated using the apparent area under the ROC curve (AUC), bootstrap optimism-corrected AUC, apparent and optimism-corrected Brier scores, net reclassification improvement (NRI), and integrated discrimination improvement (IDI). Bootstrap optimism correction was performed using 1,000 bootstrap resamples. The complete model-development procedure, including estimation of the IIDS coefficients and fitting of the sequential models, was repeated within each resample. Pairwise comparisons of the apparent AUCs of the sequential models were performed using the DeLong test.

### 2.5 Development of the final prognostic model

The final multivariable logistic regression model incorporated CCI, SOFA, and IIDS. The predicted probability of 28-day mortality was calculated as *P* = 1/[1 + exp(−*η*)], where *η* = −1.9289 + 0.3754 × CCI + 0.1013 × SOFA + 0.9000 × IIDS. Based on the final model, a nomogram was constructed to provide individualized estimates of the probability of 28-day mortality.

Internal model performance was evaluated in terms of discrimination, calibration, and clinical utility. Discrimination was assessed using the apparent ROC curve and Harrell's concordance index (C-index). Calibration was assessed using bootstrap optimism-corrected calibration plots generated from 1,000 bootstrap resamples, together with the mean absolute error (MAE). Clinical utility was evaluated using decision curve analysis (DCA).

### 2.6 Sensitivity and subgroup analyses

Four sensitivity analyses were performed to evaluate the robustness of the final prognostic model: (1) removing SOFA from the final model; (2) excluding the internally inconsistent source record identified during data verification; (3) restricting the analysis to patients without baseline immunosuppression; and (4) additionally adjusting the final model for baseline immunosuppression and corticosteroid treatment during the RICU stay.

Prespecified subgroup analyses were conducted according to age (<75 vs. ≥75 years), sex, diabetes mellitus, malignancy, and chronic lung disease. Model discrimination within each subgroup was assessed using the AUC.

### 2.7 Statistical analysis

All statistical analyses were performed using R software (version 4.5.2; R Foundation for Statistical Computing, Vienna, Austria). All tests were two-sided, and a *P* value <0.05 was considered statistically significant unless otherwise specified.

No formal *a priori* sample-size calculation was performed because this retrospective study included all consecutive patients meeting the eligibility criteria during the predefined study period.

Continuous variables are presented as mean ± standard deviation (SD) or median [interquartile range (IQR)], as appropriate, whereas categorical variables are presented as counts (percentages). Between-group comparisons were performed using the Student's *t* test or the Wilcoxon rank-sum test for continuous variables, and the Pearson chi-square test or Fisher's exact test for categorical variables, as appropriate. Comparisons of baseline characteristics were descriptive, and no adjustment for multiple testing was applied.

Discriminative performance was evaluated using receiver operating characteristic (ROC) analysis and quantified by the area under the ROC curve (AUC). Pairwise comparisons of apparent AUCs were performed using the DeLong test. Incremental predictive performance of sequential models was assessed using the net reclassification improvement (NRI), integrated discrimination improvement (IDI), and the Brier score. Internal evaluation was performed using 1,000 bootstrap resamples, with bootstrap optimism-corrected estimates reported for model discrimination, calibration, and overall model performance.

## 3 Results

### 3.1 Study population and baseline characteristics

**Figure 1:**
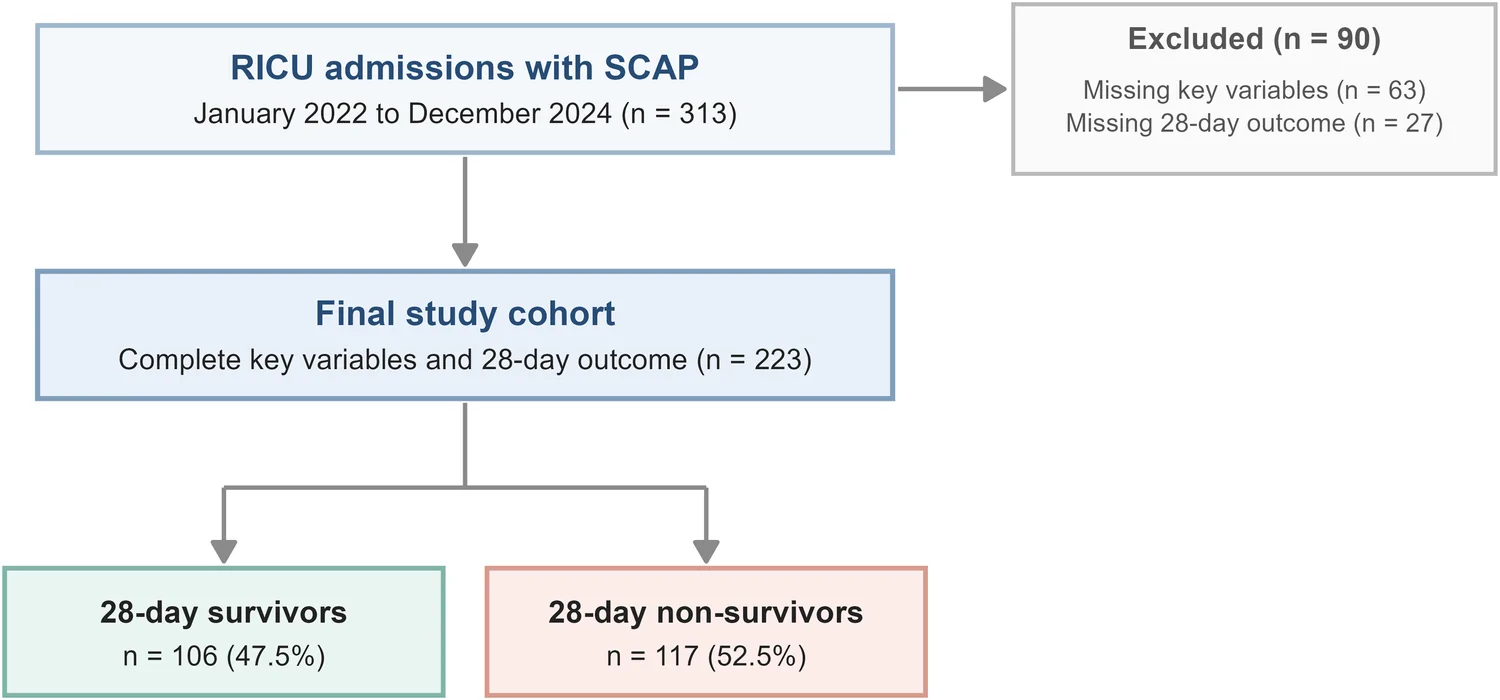
Flow diagram of patient selection and study cohort. A total of 313 consecutive patients admitted to the respiratory intensive care unit (RICU) with severe community-acquired pneumonia (SCAP) between January 2022 and December 2024 were screened. After exclusion of patients with missing key variables (*n* = 63) or unavailable 28-day outcome data (*n* = 27), 223 patients were included in the final analysis. The final cohort comprised 106 28-day survivors and 117 28-day non-survivors.

A total of 313 consecutive patients admitted to the Respiratory Intensive Care Unit (RICU) with severe community-acquired pneumonia (SCAP) were screened for eligibility. After 90 patients with missing key variables or unavailable 28-day outcome data were excluded, 223 patients were included in the final analysis (Figure 1). Of these, 106 (47.5%) were alive and 117 (52.5%) had died at 28 days after RICU admission.

Baseline characteristics are summarized in Table 1. Non-survivors had higher CCI, SOFA, and PSI scores than survivors, whereas CURB-65 scores were similar between the groups. Non-survivors also had lower CD4^+^ T-cell, CD8^+^ T-cell, NK-cell, and monocyte counts and higher NLR values. In addition, non-survivors had poorer oxygenation and higher levels of inflammatory markers.

**Table 1:** Clinical characteristics of the study cohort stratified by 28-day survival status.

Characteristic	Overall (*N* = 223)	Survivors (*n* = 106)	Non-survivors (*n* = 117)	*P* value
Demographics				
Age, years	76.0 (70.0, 83.0)	75.0 (69.0, 81.8)	78.0 (71.0, 84.0)	0.100
Male sex	173 (77.6%)	85 (80.2%)	88 (75.2%)	0.374
BMI, kg/m^2^	22.50 ± 3.89	22.32 ± 3.95	22.67 ± 3.85	0.508
Severity scores				
CCI	8 (6, 10)	7 (5, 8)	9 (8, 11)	<0.001
SOFA	6 (4, 8)	4 (3, 7)	7 (5, 9)	<0.001
PSI	162.03 ± 37.96	150.00 ± 34.77	172.93 ± 37.56	<0.001
CURB-65	3 (3, 4)	3 (3, 4)	3 (3, 4)	0.803
Immune cell profile				
CD4^+^ T cells, cells/µL	180 (88, 295)	252 (131, 341)	141 (66, 231)	<0.001
CD8^+^ T cells, cells/µL	112 (66, 210)	154 (87, 234)	95 (53, 169)	<0.001
NK cells, cells/µL	96 (40, 189)	122 (55, 224)	86 (29, 156)	0.009
B cells, cells/µL	73 (33, 135)	84 (38, 143)	63 (29, 122)	0.054
Monocyte count, ×10^9^/L	0.4 (0.2, 0.7)	0.5 (0.3, 0.8)	0.3 (0.2, 0.5)	<0.001
NLR	15.7 (9.9, 25.0)	13.5 (8.8, 19.4)	17.8 (15.1, 30.5)	<0.001
Inflammatory markers				
WBC count, ×10^9^/L	10.8 (7.4, 14.4)	11.9 (7.8, 15.2)	10.0 (6.9, 12.9)	0.017
CRP, mg/L	139.9 (75.5, 198.3)	86.7 (31.3, 150.6)	170.6 (131.0, 225.4)	<0.001
PCT, ng/mL	0.6 (0.2, 2.4)	0.6 (0.2, 2.3)	0.5 (0.2, 2.5)	0.715
Organ function				
PaO_2_/FiO_2_, mmHg	173 (102, 250)	200 (137, 285)	146 (98, 215)	<0.001
Creatinine, µmol/L	78.0 (59.0, 143.0)	76.0 (57.0, 129.0)	79.0 (60.0, 161.0)	0.154
BUN, mmol/L	9.1 (6.6, 15.1)	8.9 (6.2, 12.4)	9.8 (7.0, 15.7)	0.076
Lactate, mmol/L	1.7 (1.2, 2.5)	1.6 (1.1, 2.3)	1.8 (1.3, 2.8)	0.180
D-dimer, µg/mL	2.3 (1.3, 5.5)	2.7 (1.3, 6.2)	2.3 (1.3, 4.7)	0.678
Albumin, g/L	29.4 (26.8, 32.8)	29.6 (26.8, 33.1)	29.3 (26.8, 32.3)	0.651
Treatments during RICU stay				
Mechanical ventilation	129 (57.8%)	51 (48.1%)	78 (66.7%)	0.005
Vasopressor treatment	158 (70.9%)	53 (50.0%)	105 (89.7%)	<0.001
Corticosteroid treatment	151 (67.7%)	65 (61.3%)	86 (73.5%)	0.052
Baseline immunosuppressive conditions/exposures				
Malignancy	67 (30.0%)	23 (21.7%)	44 (37.6%)	0.010
Autoimmune disease	21 (9.4%)	9 (8.5%)	12 (10.3%)	0.652
Radiotherapy within 3 months	6 (2.7%)	1 (0.9%)	5 (4.3%)	0.216
Chemotherapy within 3 months	22 (9.9%)	9 (8.5%)	13 (11.1%)	0.512
Long-term corticosteroid exposure	22 (9.9%)	11 (10.4%)	11 (9.4%)	0.807
Non-corticosteroid immunosuppressant use	9 (4.0%)	5 (4.7%)	4 (3.4%)	0.739
Microbiological detection				
Bacterial detection	97 (43.5%)	54 (50.9%)	43 (36.8%)	0.033
Fungal detection	108 (48.4%)	50 (47.2%)	58 (49.6%)	0.720
Viral detection	99 (44.4%)	41 (38.7%)	58 (49.6%)	0.102
Atypical pathogen detection	7 (3.1%)	4 (3.8%)	3 (2.6%)	0.711

BMI, body mass index; CCI, Charlson comorbidity index; SOFA, sequential organ failure assessment; PSI, pneumonia severity index; CURB-65, confusion, urea, respiratory rate, blood pressure, age ≥65 years; NLR, neutrophil-to-lymphocyte ratio; WBC, white blood cell count; CRP, C-reactive protein; PCT, procalcitonin; PaO_2_/FiO_2_, arterial oxygen partial pressure/fraction of inspired oxygen; BUN, blood urea nitrogen.

Continuous variables are presented as mean ± standard deviation (SD) or median [interquartile range (IQR)], as appropriate. Between-group comparisons were performed using the Student's t test or the Wilcoxon rank-sum test, as appropriate. Categorical variables were compared using the Pearson chi-square test or Fisher's exact test, as appropriate. No missing values were present for variables included in Table 1. Comparisons are descriptive and were not adjusted for multiple testing.

During the RICU stay, non-survivors were more likely to require mechanical ventilation and vasopressor support, whereas corticosteroid treatment rates were similar between the groups. Malignancy was more prevalent among non-survivors, whereas the prevalence of other baseline immunosuppressive conditions and immunomodulatory exposures was similar between the groups. Bacterial pathogens were detected less frequently in non-survivors, whereas the detection rates of fungal, viral, and atypical pathogens were similar between the groups.

### 3.2 Development and discriminative performance of the immune-inflammatory dysregulation score

**Figure 2:**
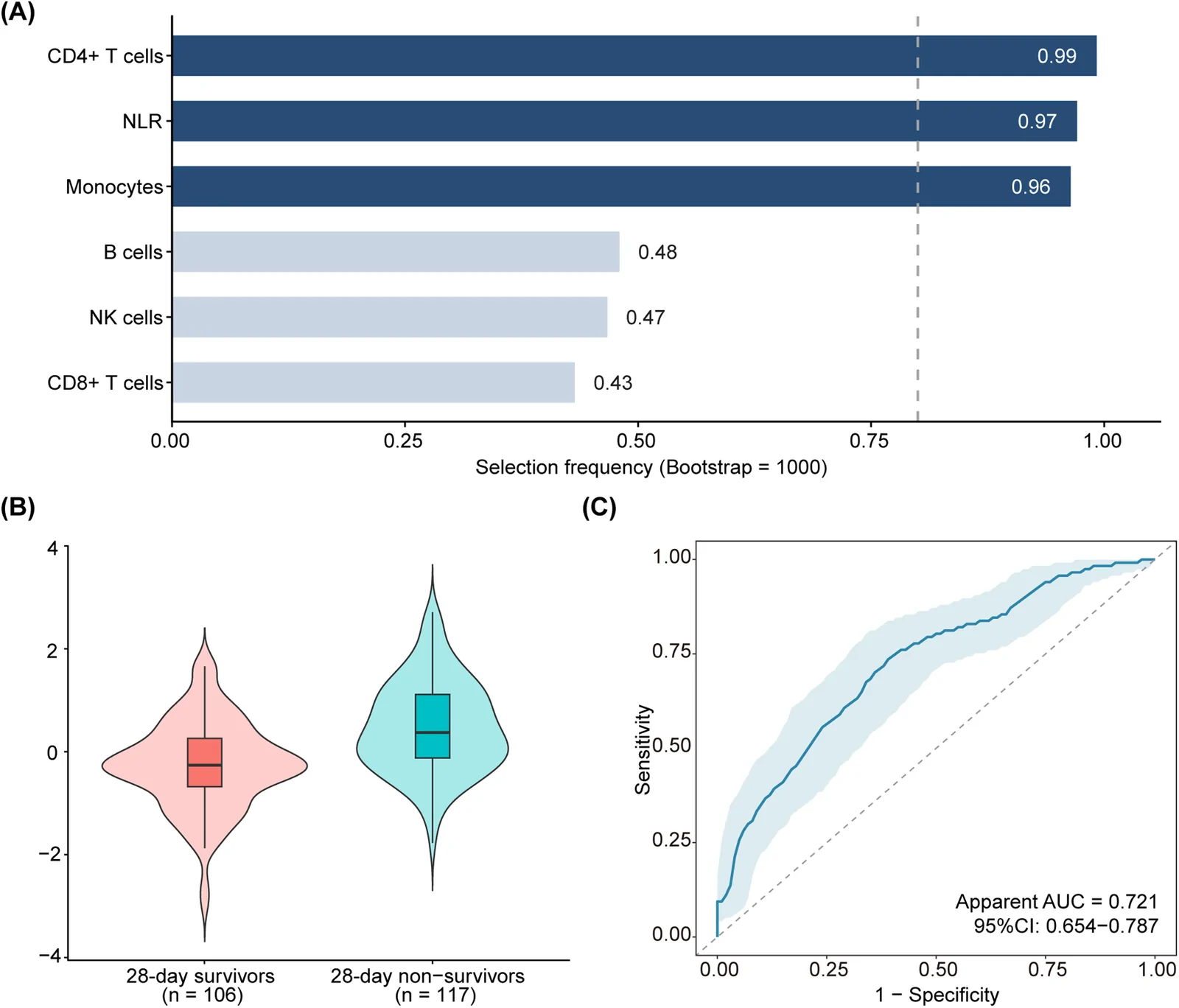
Development and apparent discriminative performance of the immune-inflammatory dysregulation score (IIDS). **(A)** Bootstrap stability selection of six prespecified immune-inflammatory biomarkers across 1,000 bootstrap resamples. Variables with a selection frequency ≥0.80 were retained for IIDS construction. **(B)** Distribution of IIDS according to 28-day survival status. Comparisons between survivors and non-survivors were performed using the Wilcoxon rank-sum test. **(C)** Receiver operating characteristic (ROC) curve evaluating the apparent discriminative performance of IIDS for predicting 28-day mortality. The apparent area under the ROC curve (AUC) was 0.721 (95% CI: 0.654–0.787).

Six prespecified immune-inflammatory biomarkers, comprising CD4^+^ T-cell count, CD8^+^ T-cell count, NK-cell count, B-cell count, monocyte count, and the neutrophil-to-lymphocyte ratio (NLR), were evaluated using bootstrap stability selection with 1,000 bootstrap resamples. Three variables, log1*p*(CD4^+^ T-cell count), log1*p*(monocyte count), and log1*p*(NLR), reached the predefined selection-frequency threshold of ≥0.80 and were retained for score construction. The remaining three biomarkers did not meet the stability criterion (Figure 2A).

The Immune-Inflammatory Dysregulation Score (IIDS) was derived from the three retained variables using multivariable logistic regression. IIDS values were higher in patients who died within 28 days than in survivors (*P* < 0.001; Figure 2B).

Receiver operating characteristic analysis yielded an apparent area under the curve (AUC) of 0.721 [95% confidence interval (CI): 0.654–0.787] for the prediction of 28-day mortality (Figure 2C).

### 3.3 Comparison of IIDS with conventional clinical severity scores

**Figure 3:**
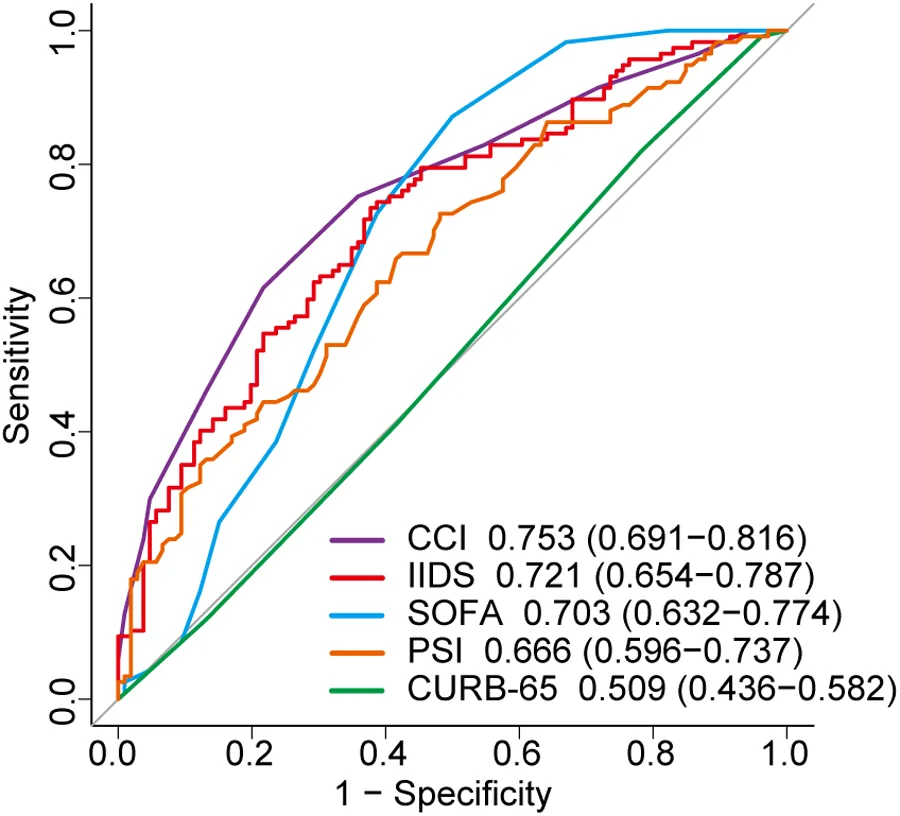
Comparison of the discriminative performance of IIDS and conventional clinical severity scores. Receiver operating characteristic (ROC) curves comparing the apparent discriminative performance of IIDS with four commonly used clinical severity scores [Charlson Comorbidity Index (CCI), Sequential Organ Failure Assessment (SOFA), Pneumonia Severity Index (PSI), and CURB-65] for predicting 28-day mortality. Areas under the ROC curves (AUCs) with 95% confidence intervals are shown in the legend. Pairwise DeLong comparisons between IIDS and each conventional clinical severity score are presented in Supplementary Table S1.

The discriminative performance of IIDS was compared with that of conventional clinical severity scores using receiver operating characteristic analysis (Figure 3). The apparent AUCs were 0.753 for CCI; 0.721 for IIDS; 0.703 for SOFA; 0.666 for PSI, and 0.509 for CURB-65.

IIDS had numerically higher apparent discrimination than SOFA, PSI, and CURB-65, whereas CCI had the highest apparent AUC among the conventional clinical scores. Pairwise DeLong tests showed no significant difference between IIDS and CCI (*P* = 0.488), SOFA (*P* = 0.703), or PSI (*P* = 0.255). IIDS showed significantly greater discrimination than CURB-65 (*P* < 0.001; Supplementary Table S1).

We next examined whether adding IIDS to conventional clinical predictors improved prognostic performance.

### 3.4 Incremental prognostic value of IIDS in sequential prediction models

**Figure 4:**
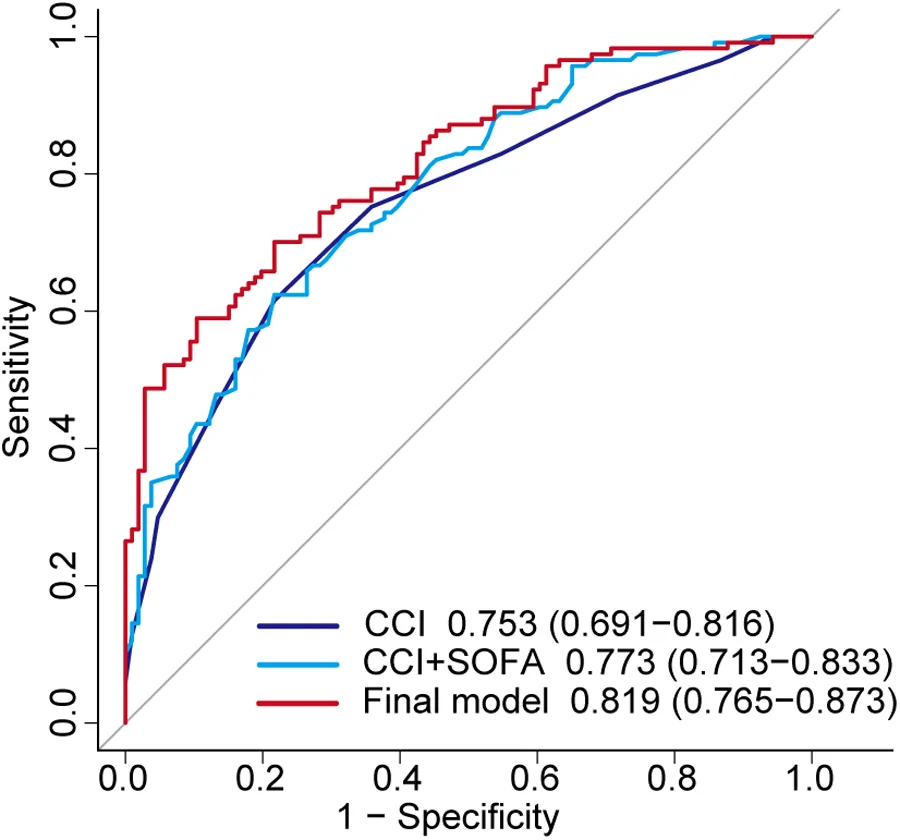
Incremental discriminative performance of sequential prediction models for 28-day mortality. Receiver operating characteristic (ROC) curves comparing sequential prediction models based on CCI alone, CCI plus SOFA, and the final model incorporating CCI, SOFA, and IIDS. Areas under the ROC curves (AUCs) with 95% confidence intervals are shown in the legend.

To evaluate the incremental prognostic contribution of IIDS beyond conventional clinical predictors, sequential prediction models were constructed by progressively adding SOFA and IIDS to the baseline CCI model (Figure 4). Discrimination increased across the sequential models, with the final model comprising CCI, SOFA, and IIDS yielding the highest apparent AUC.

Detailed performance estimates are presented in Table 2. The apparent AUC increased from 0.753 (95% CI: 0.691–0.816) for Model 1 to 0.773 (95% CI: 0.713–0.833) for Model 2 and to 0.819 (95% CI: 0.765–0.873) for Model 3. After bootstrap optimism correction, the corresponding AUCs were 0.753 (95% CI: 0.695–0.814), 0.767 (95% CI: 0.712–0.831), and 0.809 (95% CI: 0.760–0.862), respectively. Apparent Brier scores decreased across the three models, whereas the optimism-corrected Brier score of the final model was 0.218.

**Table 2:** Apparent and bootstrap optimism-corrected predictive performance of sequential prediction models for 28-day mortality. The primary bootstrap internal evaluation repeated IIDS coefficient estimation and sequential model fitting within each of 1,000 bootstrap resamples.

Model	Apparent AUC (95% CI)	Corrected AUC (95% CI)	Apparent Brier	Corrected Brier	DeLong *P* value (vs. previous model)	Apparent NRI	Corrected NRI (95% CI)	Apparent IDI	Corrected IDI (95% CI)
IIDS	0.721 (0.654–0.787)	0.708 (0.645–0.770)	NA	NA	NA	NA	NA	NA	NA
CCI	0.753 (0.691–0.816)	0.753 (0.695–0.814)	0.200	0.204	NA	NA	NA	NA	NA
CCI + SOFA	0.773 (0.713–0.833)	0.767 (0.712–0.831)	0.194	0.200	0.170	0.430	0.423 (0.166–0.698)	0.028	0.023 (−0.011–0.039)
CCI + SOFA + IIDS	0.819 (0.765–0.873)	0.809 (0.760–0.862)	0.175	0.218	0.014	0.519	0.250 (−0.226–0.686)	0.079	0.036 (−0.110–0.112)

CCI, Charlson Comorbidity Index; SOFA, Sequential Organ Failure Assessment score; IIDS, Immune-Inflammatory Dysregulation Score; NRI, net reclassification improvement; IDI, integrated discrimination improvement.
The primary bootstrap internal evaluation repeated IIDS coefficient estimation and sequential-model fitting in each of 1,000 bootstrap samples. NRI and IDI were calculated by comparing each sequential model with the model immediately preceding it. Lower Brier scores indicate better overall prediction accuracy. The full-pipeline bootstrap correction accounts for uncertainty in both IIDS coefficient estimation and sequential model fitting. No adjustment for multiple comparisons was applied to the prespecified DeLong tests.

Compared with Model 1, adding SOFA in Model 2 yielded an optimism-corrected NRI of 0.423 (95% CI: 0.166–0.698) and an optimism-corrected IDI of 0.023 (95% CI: −0.011 to 0.039). Compared with Model 2, adding IIDS in Model 3 yielded an optimism-corrected NRI of 0.250 (95% CI: −0.226 to 0.686) and an optimism-corrected IDI of 0.036 (95% CI: −0.110 to 0.112). The apparent AUC increased after adding IIDS to the CCI + SOFA model (DeLong *P* = 0.014), although the confidence intervals for the optimism-corrected NRI and IDI included zero.

### 3.5 Development and internal evaluation of the final prognostic model

The final multivariable logistic regression model incorporated CCI, SOFA, and IIDS (Table 3). After adjustment, higher CCI [odds ratio (OR): 1.46; 95% CI: 1.26–1.69; *P* < 0.001] and higher IIDS (OR: 2.46; 95% CI: 1.63–3.70; *P* < 0.001) were independently associated with greater odds of 28-day mortality. SOFA did not reach conventional statistical significance after adjustment (OR: 1.11; 95% CI: 0.99–1.24; *P* = 0.072) but was retained in accordance with the prespecified sequential modeling strategy because it is an established measure of acute organ dysfunction.

**Table 3:** Multivariable logistic regression model for 28-day mortality IIDS was entered into the model as a continuous linear predictor derived from log1p-transformed immune biomarkers.

Predictor	*β* coefficient	Adjusted OR	95% CI	*P* value
CCI	0.375	1.46	1.26–1.69	<0.001
SOFA	0.101	1.11	0.99–1.24	0.072
IIDS	0.900	2.46	1.63–3.70	<0.001
Intercept	−1.929	NA	NA	NA

CCI, Charlson Comorbidity Index; SOFA, Sequential Organ Failure Assessment score; IIDS, Immune-Inflammatory Dysregulation Score.
Adjusted odds ratios are reported per one-unit increase in each predictor. IIDS = −0.5475 × log1*p*(CD4^+^ T-cell count) − 1.8078 × log1*p*(monocyte count) + 0.5915 × log1*p*(NLR) SOFA was retained in the final model based on the prespecified sequential modeling strategy despite not reaching conventional statistical significance.

**Figure 5:**
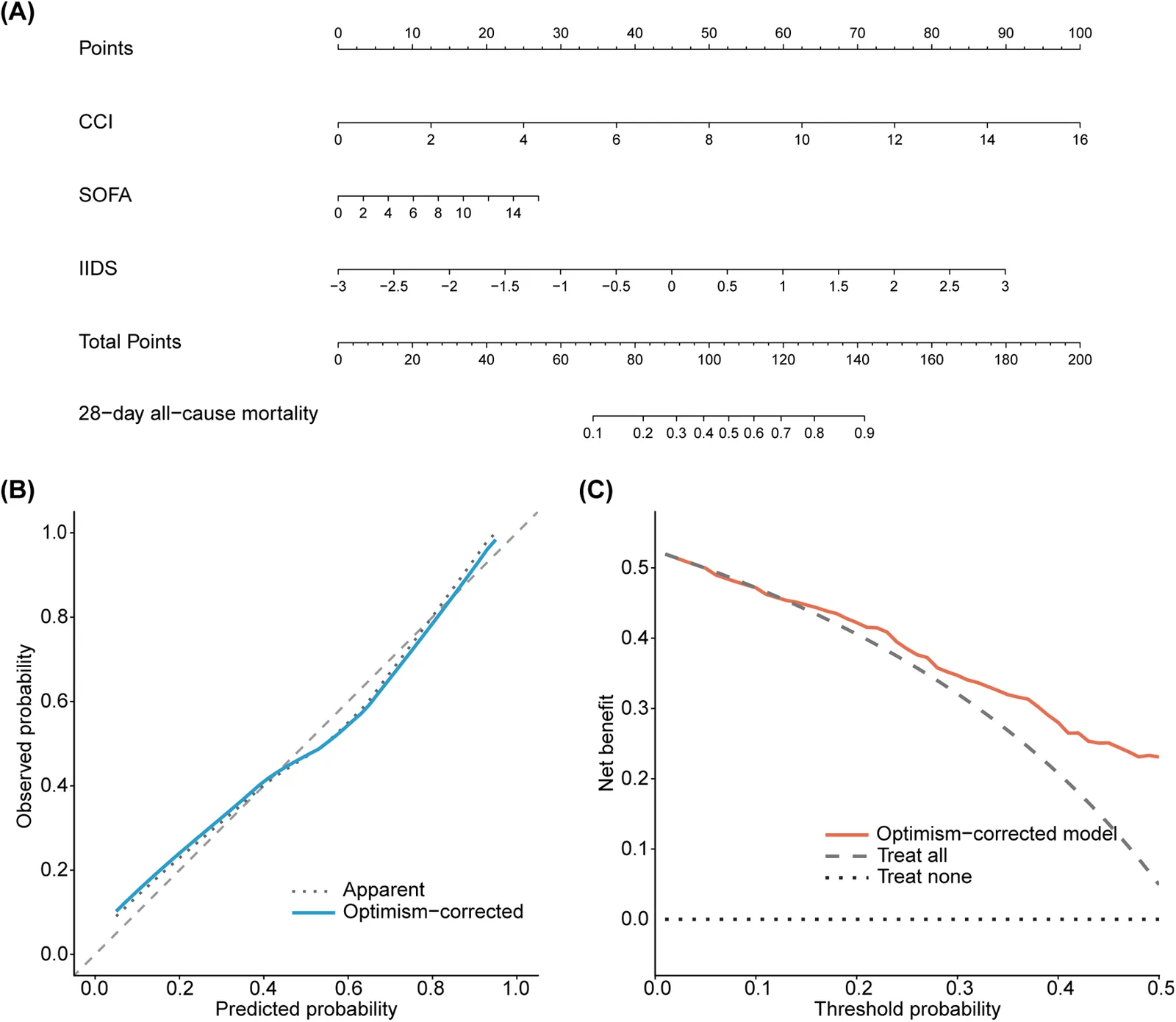
Nomogram and internal evaluation of the final prognostic model. **(A)** Nomogram developed from the final multivariable logistic regression model incorporating CCI, SOFA, and IIDS for individualized prediction of 28-day mortality. **(B)** Calibration plot comparing predicted and observed probabilities of 28-day mortality. The apparent calibration curve and bootstrap optimism-corrected calibration curve are shown together with the reference line representing perfect calibration. **(C)** Decision curve analysis evaluating the clinical utility of the optimism-corrected final model. Net benefit across a range of threshold probabilities is compared with the default strategies of treating all patients or treating none.

Based on the final model, a nomogram was constructed to provide individualized estimates of the probability of 28-day mortality (Figure 5A). After bootstrap optimism correction, the calibration curve showed close agreement between predicted and observed event probabilities and remained close to the apparent calibration curve and reference line across the evaluated risk range (Figure 5B).

Decision curve analysis indicated that the final model had potential clinical utility. Across the evaluated range of threshold probabilities, the optimism-corrected model provided a greater net benefit than the default strategies of treating all patients or treating none (Figure 5C).

### 3.6 Sensitivity and subgroup analyses

The final model showed similar performance across the four sensitivity analyses (Table 4). Removing SOFA resulted in a modest reduction in discrimination (AUC: 0.812 vs. 0.819 in the primary analysis), whereas exclusion of the flow-cytometry record identified during data verification yielded an AUC of 0.825. Restricting the analysis to patients without baseline immunosuppression yielded an AUC of 0.826, and additional adjustment for baseline immunosuppression and corticosteroid treatment during the RICU stay yielded an AUC of 0.817. IIDS remained independently associated with 28-day mortality in all sensitivity analyses, with adjusted ORs ranging from 2.37 to 3.23 (all *P* < 0.001).

**Table 4:** Sensitivity analyses of the final prognostic model.

Sensitivity analysis	N	Deaths	AUC	Brier	IIDS β	IIDS OR (95% CI)	*P* value
Primary model (CCI + SOFA + IIDS)	223	117	0.819	0.175	0.900	2.46 (1.63–3.70)	<0.001
Model without SOFA: CCI + IIDS	223	117	0.812	0.177	0.959	2.61 (1.74–3.90)	<0.001
Exclusion of the flow-cytometry record identified during data verification; IIDS re-derived	222	116	0.825	0.172	0.918	2.50 (1.66–3.77)	<0.001
Exclude baseline immunosuppression; primary IIDS retained	136	63	0.826	0.169	1.173	3.23 (1.81–5.76)	<0.001
Additional adjustment for baseline immunosuppression and in-RICU corticosteroid treatment	223	117	0.817	0.175	0.865	2.37 (1.56–3.61)	<0.001

CCI, Charlson Comorbidity Index; SOFA, Sequential Organ Failure Assessment score; IIDS, Immune-Inflammatory Dysregulation Score. Analyses address SOFA retention, the internally inconsistent flow-cytometry record, and baseline or treatment-related immunosuppression.
The flow-cytometry record identified during data verification was retained in the primary analysis because the value in the analytical database matched the original laboratory report. Its exclusion was evaluated in a sensitivity analysis. Baseline immunosuppression was defined by malignancy, autoimmune disease, recent radiotherapy or chemotherapy, long-term corticosteroid exposure, or non-corticosteroid immunosuppressant use. Corticosteroid treatment after RICU admission was included only in a sensitivity adjustment because it is a post-baseline treatment exposure.

**Figure 6:**
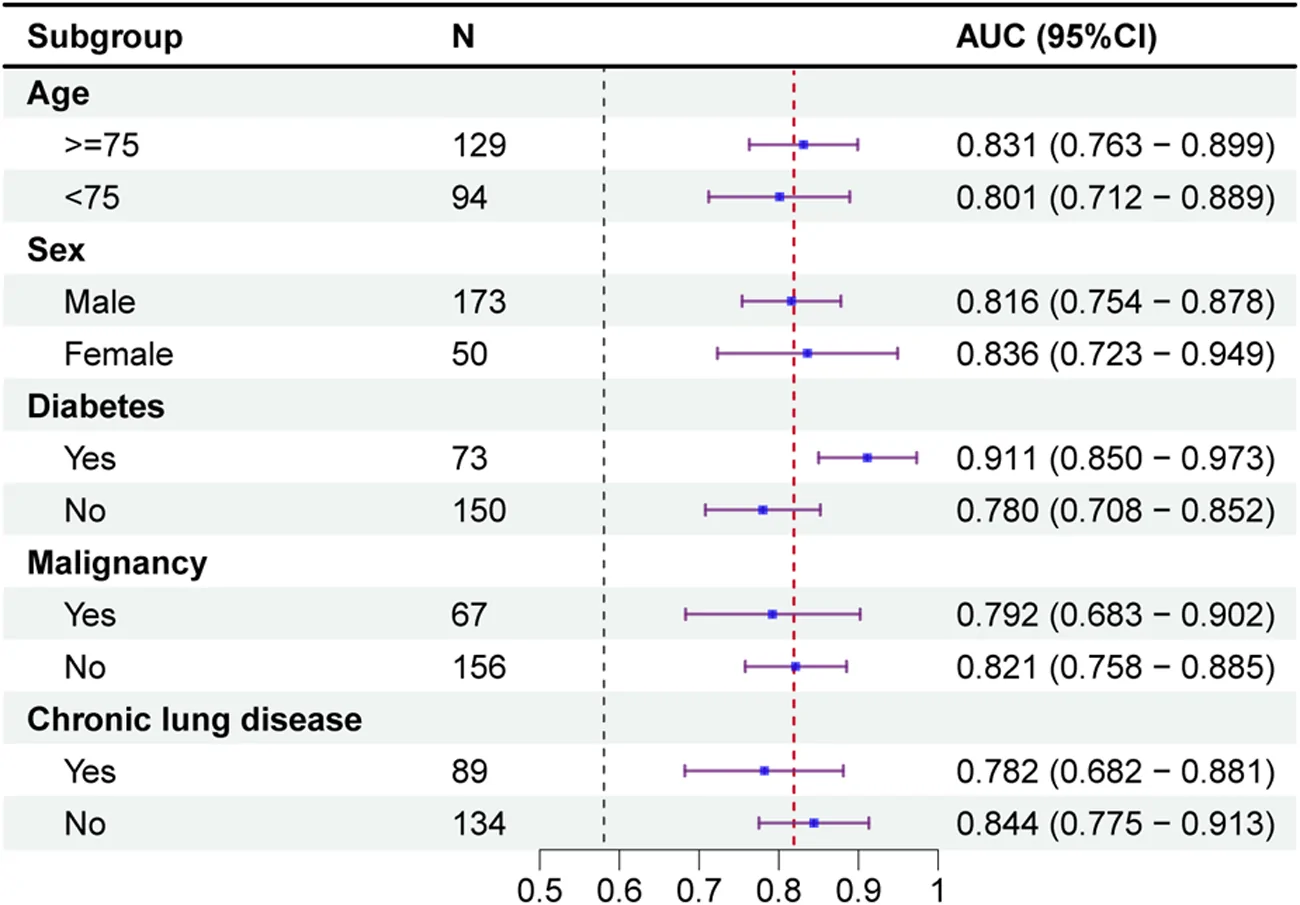
Subgroup analyses of the discriminative performance of the final prognostic model. Forest plot showing the area under the receiver operating characteristic curve (AUC) and corresponding 95% confidence interval for the final model across prespecified clinical subgroups stratified by age (<75 or ≥75 years), sex, diabetes, malignancy, and chronic lung disease.

Subgroup analyses showed broadly similar discrimination across groups defined by age, sex, diabetes mellitus, malignancy, and chronic lung disease (Figure 6). Subgroup-specific AUC estimates were generally comparable.

## 4 Discussion

In this retrospective cohort of critically ill patients with severe community-acquired pneumonia (SCAP), we developed and internally evaluated the Immune-Inflammatory Dysregulation Score (IIDS), an immune-informed prognostic signature comprising CD4^+^ T-cell count, monocyte count, and the neutrophil-to-lymphocyte ratio (NLR). Although IIDS alone showed discrimination comparable to that of several established clinical severity scores, its principal contribution was the additional prognostic information it provided beyond conventional clinical predictors. Adding IIDS to a sequential model containing the Charlson Comorbidity Index (CCI) and Sequential Organ Failure Assessment (SOFA) score improved apparent discrimination, while the optimism-corrected AUC remained higher than those of the simpler sequential models; however, optimism-corrected reclassification estimates were imprecise and their confidence intervals included zero. The final model showed close agreement between predicted and observed risks after bootstrap optimism correction and provided greater net benefit than the default strategies across the evaluated threshold-probability range. Its discrimination was also broadly similar across the sensitivity analyses and clinically relevant subgroups examined. Together, these findings suggest that host immune status may represent a dimension of SCAP severity that is not directly captured by conventional clinical assessment.

The biological rationale for IIDS is supported by evidence that severe pneumonia involves concurrent inflammatory activation and impaired host immunity (5, 7, 8, 18). Recent immune-profiling and single-cell transcriptomic studies have identified extensive remodeling of lymphoid and myeloid-cell states, including lymphocyte depletion, T-cell exhaustion, altered monocyte phenotypes, and expansion of immunosuppressive myeloid-cell populations in severe disease (7, 8, 18). In clinical CAP cohorts, depletion of CD4^+^ T-cell subsets has been associated with greater disease severity, delayed recovery, and mortality (4–7). Monocytes are central to innate host defense, and changes in their abundance and functional state may reflect the balance between inflammatory activation and immune suppression during severe pneumonia (8, 18). NLR similarly captures the balance between circulating neutrophils and lymphocytes and has been associated with adverse outcomes in CAP, although its incremental prognostic value beyond established clinical scores has varied across cohorts (14–16).

By combining markers of adaptive immunity, innate immunity, and systemic inflammation, IIDS was designed to capture complementary components of the host response that are incompletely represented by any single biomarker. Contemporary immune-stratification approaches have derived immune-dysregulation states from broad plasma-biomarker panels and machine-learning models (18). In contrast, IIDS uses hematological and lymphocyte-subset measurements already collected in our RICU, which may offer a more accessible framework in centers where lymphocyte-subset testing is available. Its practical value and transportability, however, require confirmation in independent external cohorts before clinical implementation.

An important implication of our findings is that IIDS should complement, rather than replace, established clinical severity scores. Among the conventional predictors evaluated in our cohort, CCI had the highest apparent discriminative performance. This finding may reflect the advanced age, substantial comorbidity burden, and high illness severity of patients admitted to our RICU. Comorbidities are important determinants of mortality in hospitalized CAP (19), and contemporary ICU cohorts show substantial variation in patient characteristics, organ-support requirements, and mortality (3, 20). Such variation is influenced partly by differences in case mix and ICU admission practices across healthcare systems. Our cohort therefore represents a high-risk SCAP population in which chronic comorbidity burden may strongly influence physiological reserve and the capacity to withstand severe infection. This may help explain why CCI remained the strongest conventional predictor across the sequential models.

In contrast, PSI and CURB-65 showed limited discrimination in our cohort. This finding should not be interpreted as evidence against their established clinical value because both scores were developed for risk stratification in broader CAP populations rather than exclusively in critically ill patients requiring intensive care (12, 13). A systematic review of hospitalized CAP populations reported summary AUCs of approxima tely 0.81 for PSI and 0.80 for CURB-65 in predicting mortality (21). By contrast, a meta-analysis focused on identifying patients requiring intensive care reported an AUC of approximately 0.69 for both scores (22). Their lower discrimination in advanced disease may partly reflect the narrower clinical spectrum of an RICU cohort, in which many patients already meet high-risk criteria. Conventional scores based predominantly on demographics, comorbidities, physiological abnormalities, and organ dysfunction may therefore provide less differentiation among patients with established critical illness and do not directly characterize variation in host immune status.

Although CD8^+^ T-cell and NK-cell counts differed between survivors and non-survivors, neither variable met the predefined bootstrap stability-selection threshold. This does not exclude their biological or prognostic relevance. Severe pneumonia is associated with broad alterations in CD8^+^ T-cell, NK-cell, and other lymphocyte populations (5, 7, 8), but the incremental predictive contribution of an individual marker may vary across resampled datasets. In the present analysis, CD4^+^ T-cell count, monocyte count, and NLR were selected more consistently than CD8^+^ T-cell and NK-cell counts. The bootstrap-based strategy therefore favored variables with more stable predictive contributions in this cohort, yielding a more parsimonious immune signature while reducing sensitivity to sampling variation.

This study has several strengths. First, IIDS combines a routinely derived hematological index with immune-cell measurements available in our RICU and does not require newly developed molecular assays. Second, IIDS was designed to complement rather than replace conventional severity scores, and its incremental prognostic contribution was quantified using a prespecified sequential modeling strategy. Third, bootstrap-based stability selection was combined with bootstrap optimism correction, reducing susceptibility to overfitting and providing more realistic estimates of internal model performance. Finally, the resulting model showed close agreement between predicted and observed risks after optimism correction, provided potential net clinical benefit across the evaluated threshold-probability range, and was presented as a nomogram to support individualized risk estimation.

Several limitations should be acknowledged. First, this was a retrospective, single-center study, which limits the transportability of the findings. Although bootstrap optimism correction was applied throughout model development and internal evaluation, the model has not been externally validated. Independent validation in multicenter cohorts is therefore required before clinical implementation. Second, 90 of the 313 screened patients (28.8%) were excluded because key variables were missing or 28-day outcome data were unavailable. Complete-case inclusion may have introduced selection bias, particularly because the availability of lymphocyte-subset measurements may have been influenced by clinical practice. Third, immune biomarkers were assessed only within the first 24 h after RICU admission, precluding evaluation of longitudinal changes in immune status and their relationship with treatment response or disease progression. Fourth, immune alterations related to chronic comorbidities, malignancy, previous treatment, or other immunocompromising conditions could not be fully distinguished from those induced by acute infection. Although IIDS remained associated with 28-day mortality across the sensitivity analyses, residual confounding and instability arising from the modest sample size cannot be excluded.

Overall, we developed and internally evaluated an immune-informed model incorporating IIDS for prediction of 28-day mortality in critically ill patients with SCAP. By integrating measures related to adaptive immunity, innate immunity, and systemic inflammation, IIDS provided prognostic information complementary to conventional clinical severity scores and improved the internally evaluated performance of sequential prediction models. Multicenter external validation is now required to assess model transportability, investigate longitudinal immune dynamics, and determine whether immune-informed risk stratification can support clinical decision-making and improve patient outcomes.

## 5 Conclusion

The Immune-Inflammatory Dysregulation Score (IIDS) provided prognostic information complementary to conventional clinical predictors in critically ill patients with severe community-acquired pneumonia. Adding IIDS to a sequential prediction model improved apparent discrimination when added to conventional clinical predictors, although the uncertainty in optimism-corrected reclassification estimates underscores the need for independent external validation. The resulting nomogram may support individualized risk estimation, but independent external validation is required before its routine clinical implementation.

## Data Availability

The raw data supporting the conclusions of this article will be made available by the authors, without undue reservation.
