# Supplementary material for: An immune-inflammatory dysregulation score for risk stratification of 28-day all-cause mortality in critically ill patients with severe community-acquired pneumonia
Source: Front Med (Lausanne). 2026 Sep 1;13:1923412. doi: 10.3389/fmed.2026.1923412 (PMC13574933; doi:10.3389/fmed.2026.1923412)
Supplement: Supplementary file 1 [file Table1.docx]

Supplementary Material

# 1 Supplementary Figures and Tables

## 1.1 Supplementary Table

**Supplementary Table S1. Pairwise DeLong comparisons of IIDS with conventional clinical severity scores**

| Comparison | AUC difference | Z statistic | P value |
| --- | --- | --- | --- |
| IIDS vs CCI | −0.033 | -0.694 | 0.488 |
| IIDS vs SOFA | 0.018 | 0.381 | 0.703 |
| IIDS vs PSI | 0.054 | 1.139 | 0.255 |
| IIDS vs CURB-65 | 0.211 | 4.508 | <0.001 |

**Abbreviations:** CCI, Charlson Comorbidity Index; SOFA, Sequential Organ Failure Assessment score; IIDS, Immune-Inflammatory Dysregulation Score; PSI, Pneumonia Severity Index; CURB-65, Confusion, Urea, Respiratory rate, Blood pressure, age ≥65 years.

**Notes**

Two-sided paired DeLong tests were performed using the same study cohort (n = 223). P values are unadjusted because these comparisons were prespecified.
